# Blood pressure variability and medial temporal atrophy in apolipoprotein ϵ4 carriers

**DOI:** 10.1007/s11682-021-00553-1

**Published:** 2021-09-28

**Authors:** Isabel J. Sible, Daniel A. Nation, for the Alzheimer’s Disease Neuroimaging Initiative

**Affiliations:** 1 Department of Psychology, University of Southern California, Los Angeles, CA 90007 USA; 2 Institute for Memory Impairments and Neurological Disorders, University of California Irvine, Irvine, CA 92697 USA; 3 Department of Psychological Science, University of California Irvine, Irvine, CA 92697 USA

**Keywords:** Blood pressure variability; Medial temporal lobes; Apolipoprotein ϵ4; Alzheimer’s disease; Biomarkers

## Abstract

Blood pressure variability is an emerging risk factor for dementia but relationships with markers of neurodegeneration and Alzheimer’s disease risk are understudied. We investigated blood pressure variability over one year and follow-up medial temporal brain volume change in apolipoprotein ϵ4 carriers and non-carriers, and in those with and without Alzheimer’s disease biomarker abnormality. 1051 Alzheimer’s Disease Neuroimaging Initiative participants without history of dementia or stroke underwent 3–4 blood pressure measurements over 12 months and ≥ 1 MRI thereafter. A subset (*n* = 252) underwent lumbar puncture to determine Alzheimer’s disease cerebral spinal fluid amyloid-beta and phosphorylated tau biomarker abnormality. Blood pressure variability over 12 months was calculated as variability independent of mean. Longitudinal hippocampal and entorhinal cortex volume data were extracted from serial brain MRI scans obtained after the final blood pressure measurement. Apolipoprotein ϵ4 carrier status was defined as at least one ϵ4 allele. Bayesian growth modelling revealed a significant interaction of blood pressure variability by ϵ4 by time on hippocampal (ß: -2.61 [95% credible interval -3.02, -2.12]) and entorhinal cortex (ß: -1.47 [95% credible interval -1.71, -1.17]) volume decline. A similar pattern emerged in subsets with Alzheimer’s disease pathophysiology (i.e., abnormal levels of both amyloid-beta and phosphorylated tau). Findings suggest that elevated blood pressure variability is related to medial temporal volume loss specifically in ϵ4 carriers, and in those with Alzheimer’s disease biomarker abnormality. Findings could implicate blood pressure variability in medial temporal neurodegeneration observed in older ϵ4 carriers and those with prodromal Alzheimer’s disease.

**Supplementary Information:**

The online version contains supplementary material available at 10.1007/s11682-021-00553-1.

## Introduction

Blood pressure (BP) is among the most studied vascular risk factors linked to cognitive impairment, neuropathological change, and dementia (Lane et al., 2019; Zlokovic, 2011). There has been substantial work to determine the relationships between both high and low BP and brain pathology and cognitive outcomes, as well as the aggregate impact of dysregulated BP on later brain health (Lane et al., 2019). A recent randomized controlled trial found that aggressive BP lowering was related to a decreased incidence of cognitive impairment (Wright et al., 2015), suggesting a causal association between average BP levels and cognitive decline (Yaffe, 2019).

Beyond average levels, BP variability (BPV) over months and years represents an understudied aspect of BP as it relates to brain health (Yoo et al., 2020). Given the substantial overlap between vascular and Alzheimer’s disease (AD) pathologies in the brains of individuals diagnosed with dementia (Schneider et al., 2007), there is growing interest to study BPV in the context of cognitive aging and AD risk. Recent work suggests that elevated BPV is associated with cerebrovascular disease (Ma, et al., 2020a) and predictive of cognitive impairment and dementia, including AD and vascular dementia (Ma et al., 2020b; Rouch et al., 2020; Yoo et al., 2020), even in healthy older adults with well-controlled average BP (Cho et al., 2018). Chronic large fluctuations in BP may stress arterial walls and promote microvascular injury and arterial remodeling (Nagai et al., 2017). These vascular changes may convey vulnerability to cerebral hypoperfusion injury (Sible et al., 2021b) and subsequent neuronal injury, especially in regions highly sensitive to BP-related hypoxic-ischemic injury, such as the hippocampus (Iadecola, 2004; Ma et al,. 2020b; Vikner et al., 2021).

Consistent with this hypothesis, higher BPV has been linked to lower hippocampal volume in both cross-sectional (Sabayan et al., 2013) and longitudinal (Ma, et al. 2020b) studies of older adults without dementia. However, less is known about the relationship between BPV and brain volumes in other key regions of AD, such as the entorhinal cortex. It is also unclear how BPV may be related to medial temporal atrophy rates in those at risk for AD due to the presence of the apolipoprotein ϵ4 (APOE ϵ4) gene, which has been associated with neurodegeneration and neurovascular deficits in the hippocampus and parahippocampal cortex (Burggren et al., 2008; Palop & Mucke, 2011). Finally, while most studies of BPV in aging have drawn from samples clinically determined to be without history of dementia, a recent study found that BPV was increased in individuals with mild cognitive impairment (MCI) and AD biomarker abnormality (Sible et al., 2020). How these relationships may appear in samples with abnormal AD biomarkers is less known, and could have implications for neurodegeneration in AD. The aims of the present study were to investigate the interactive relationship between BPV and APOE ϵ4 carrier status in relation to hippocampal and entorhinal cortex volumetric change in older adults at risk for AD, and to determine whether these relationships remained evident in those with ongoing AD pathophysiology (i.e., AD biomarker abnormality).

## Methods

### Study design

#### Participants

Data were obtained from the Alzheimer’s Disease Neuroimaging Initiative (ADNI) database. The ADNI is a multisite natural history study that has collected clinical, biomarker, and neuropsychological data since 2003 to measure the progression of typical aging, MCI, and AD. Adults aged 55–91 were enrolled if they met the following criteria: few depressive symptoms (Geriatric Depression Scale < 6), free of history of neurological disease (other than suspected AD), no greater than mild dementia symptoms (Clinical Dementia Rating scale ≤ 1), and low vascular risk (Hachinski Ischemic Score ≤ 4). Ethical approval was obtained for each institution involved and all participants provided written informed consent. Further study details can be found online (https://adni.loni.usc.edu).

The present study included participants who underwent clinical evaluation at study baseline and BP measurement at study screening, baseline, and 6- and 12- months follow-up. Participants also underwent ≥ 1 structural MRI after the 12-month follow-up BP collection. A subset underwent lumbar puncture to determine cerebral spinal fluid (CSF) AD biomarker levels.

### Measures

#### Clinical assessment

Baseline clinical evaluation identified participants to be cognitively normal (CN) or MCI. All participants were without history of dementia or stroke. MCI diagnostic criteria included (Petersen et al., 2010): subjective memory complaint; Mini Mental State Exam scores between 24 and 30 (inclusive); global Clinical Dementia Rating scale score of 0.5; scores on delayed recall of Story A of the Wechsler Memory Scale Revised Logical Memory II subtest that are below expected performance based on years of education; general presentation that would disqualify for a diagnosis of AD. Participants were categorized as CN if diagnostic criteria for MCI were not met. CN and MCI participants were then collapsed into one category of older adults without history of dementia or stoke and were used in all analyses.

#### CSF AD biomarker assessment

Baseline lumbar puncture and CSF analysis in a subset of participants determined amyloid-beta (Aβ) and phosphorylated tau (Ptau) levels as described elsewhere (Bittner et al., 2016; Hansson et al., 2018; Seibyl et al., 2017; Shaw et al., 2016). Using established guidelines, CSF Aβ levels ≤ 980 pg/mL and CSF Ptau levels ≥ 21.8 pg/mL were considered abnormal (Hansson et al., 2018; Shaw et al., 2018).

Participants were then further categorized based on abnormal levels of both Aβ and Ptau (Aβ + Ptau +), thus representing a group of older adults without a history of dementia or stroke confirmed to have AD pathophysiology (Jack et al., 2018). To examine associations in subsets not meeting biomarker criteria for AD (e.g., Aβ + Ptau +), remaining participants with available CSF biomarker data were then categorized as those with 1) one abnormal biomarker and one normal biomarker (Aβ + Ptau- or Aβ-Ptau +); or 2) two normal biomarkers (Aβ-Ptau-) (see Supplementary Table 1).

#### BP assessment

Seated BP measurements were obtained from participants 3–4 times between study screening and 12-months follow-up using a calibrated mercury sphygmomanometer, as described elsewhere (Sible et al., 2020, 2021b). Intraindividual BPV was calculated for each participant using the 3–4 BP measurements collected over the 12 month period as variation independent of mean (VIM), a commonly used index of visit-to-visit BPV that is uncorrelated with average BP levels across visits (de Heus et al., 2019; Rothwell et al., 2010; Rouch et al., 2020; Sible et al., 2020, 2021a, 2021b). VIM was calculated as: VIM = SD/mean^*x*^, where the power *x* was derived from non-linear curve fitting of BP SD against average BP using the nls package in R (R Core Team, 2018), as described elsewhere (Rothwell et al., 2010; Yano, 2017). Baseline hypertension was determined from the total sample average systolic BP taken at study baseline. Given that systolic BPV and diastolic BPV were correlated and primary findings were similar, main findings focused on systolic BPV and diastolic BPV findings are reported in Supplementary Materials.

#### Volumetric MRI change assessment

Participants underwent ≥ 1 1.5 T or 3 T MRI after the final BP collection at 12-months follow-up. Image acquisition and processing details can be found online (http://adni.loni.usc.edu/methods/documents/mri-protocols/). Briefly, T1-weighted structural images were collected using either a 3D-MPRAGE or 3D IR-SPGR sequence. The following values from each of these MRI scans were extracted from the adnimerge dataset (Fischl, 2012; Reuter et al., 2012): total hippocampal volume, total entorhinal cortex volume, whole brain volume (sum of gray matter and white matter volumes), and total intracranial volume (TIV; sum of gray matter, white matter and CSF volumes). Volumes were determined using the FreeSurfer imaging suite as described elsewhere (http://adni.loni.usc.edu/methods/), a software with good test–retest reliability for volumetric segmentation within and across scanners (Brown et al., 2020).

#### Other measurements

Demographic and clinical information was determined from baseline clinical evaluation. Baseline body mass index (BMI) was calculated as weight (kg) / height (meters) squared. Determination of APOE ϵ4 carrier status was performed as previously described (Saykin et al., 2010) using blood samples from baseline venipuncture and participants were categorized as those with at least one APOE ϵ4 allele versus those without. Vascular risk was determined from baseline clinical evaluation, as described elsewhere (D’Agostino et al., 1994; Nation et al., 2015; Sible et al., 2020). Participants were categorized as having low (≤ 1 vascular risk factor) or high (≥ 2 vascular risk factors) vascular risk (D’Agostino et al., 1994). History of smoking and dyslipidemia were also determined from clinical evaluation at baseline. Information about medication use was determined at study baseline. Participants were categorized as those taking antihypertensive medication (all classes) versus those who were not, and those taking antidementia agents versus those who were not.

## Statistical analysis

Bayesian linear growth modelling using the brms package in R (R Core Team, 2018) examined the role of BPV, APOE ϵ4 carrier status, and the passage of time on volumetric change in hippocampus and entorhinal cortex. Compared to repeated measures ANOVA, Bayesian linear growth modelling handles missing participant data and thus boosts statistical power, as well as accommodates varying time windows of measurement. All models specified random intercepts for participant, to account for individual variation in volumetric change, and fixed effects for BPV and APOE ϵ4 carrier status to test for differences in volumetric change due to BPV and APOE ϵ4 carrier status, respectively. Only MRIs acquired after the final BP measurement at 12-months follow-up were used in analyses to determine temporal order of any associations; therefore, passage of time for MRIs was calculated as months elapsed since BPV determination (e.g., after the last BP measurement was collected at 12-months follow-up) (range: 6 – 108 months) and then grand centered at 0. In an attempt to replicate a previous finding linking BPV to hippocampal volume decline (Ma et al., 2020c), we first ran models investigating a BPV by time interaction on volumetric change in the hippocampus and entorhinal cortex. Next, because we expected volumetric change might have different trajectories based on both BPV (Ma et al., 2020c) and APOE ϵ4 carrier status (Palop & Mucke, 2011), we additionally tested the interaction of BPV by APOE ϵ4 carrier status by time. We also examined the three-way interaction of BPV by APOE ϵ4 carrier status by time on volumetric change in exploratory post-hoc analyses of subsets not meeting biomarker criteria for AD (see Supplementary Table 2). Potential confounding variables were included in all models: age at MRI, sex, years of education, APOE ϵ4 carrier status (for main effect models), TIV at MRI, baseline hypertension, antihypertensive medication use and vascular risk. Additional sensitivity analyses covaried for: 1) average BP, 2) history of smoking, 3) history of dyslipidemia, 4) use of antidementia agents, and 5) whole brain volume at MRI (instead of TIV). Supplementary analyses examined relationships between BPV and whole brain volume at MRI. All analyses were 2-tailed and effect estimates with credible intervals excluding 0 were considered significant.

## Results

In primary analyses, 1051 participants without a history of dementia or stroke contributed to 2656 MRI scans (median 3 scans) and the median time interval between BPV measurement and MRI scan was 24 months (IQR: 30 months). In secondary analyses, 252 participants confirmed to have AD pathophysiology contributed to 595 MRI scans (median 3 scans) and the median time interval between BPV measurement and MRI scan was 12 months (IQR: 30 months). Table 1 and Supplementary Table 3 summarize baseline demographic and clinical information.

**Table 1:** Baseline clinical and demographic information

	Total sample (*N* = 1051)
Age (years)	73.7 (6.8)
Sex (*n*, % female)	455 (43.3%)
Education (years)	16.0 (2.8)
APOE ϵ4 carriers (*n*, %)	437 (41.6%)
MCI (*n*, %)	680 (64.7%)
Aβ (n, % abnormal)	438 (41.7%)
Ptau (n, % abnormal)	431 (41.0%)
MMSE score	28.1 (1.7)
CDR-sb score	0.96 (0.96)
BMI (kg/m^2^)	27.0 (4.5)
Vascular risk (*n*, % low)	978 (93.1%)
Medication use (*n*, %)	
Antihypertensive agents	439 (41.8%)
ACE inhibitors	181 (17.2%)
ARBs	96 (9.1%)
Alpha blockers	24 (2.3%)
Calcium channel blockers	82 (7.8%)
Diuretics	56 (5.3%)
Antidementia agents	398 (37.9%)
Systolic BP (mmHg)	
Baseline	134.6 (16.9)
Average	133.2 (13.5)
VIM	5.3 (3.6)
Diastolic BP (mmHg)	
Baseline	74.6 (9.8)
Average	73.6 (7.8)
VIM	5.9 (1.2)

Means and SDs shown unless otherwise indicated

*MMSE*,  Mini Mental State Exam; *BP*, blood pressure; *BMI*, body mass index: *VIM,* variability independent of mean; *APOE ϵ4*,  apolipoprotein ϵ4; *MCI*, mild cognitive impairment; *CDR-sb*, Clinical Dementia Rating Scale sum of box score; *Aβ*, amyloid-beta; *Ptau*, phosphorylated tau; *ACE inhibitors*, angiotensin-converting enzyme inhibitors; *ARBs*, angiotensin II receptor blockers

### BPV and APOE ϵ4 related to medial temporal volumetric change in older adults

**Fig. 1:**
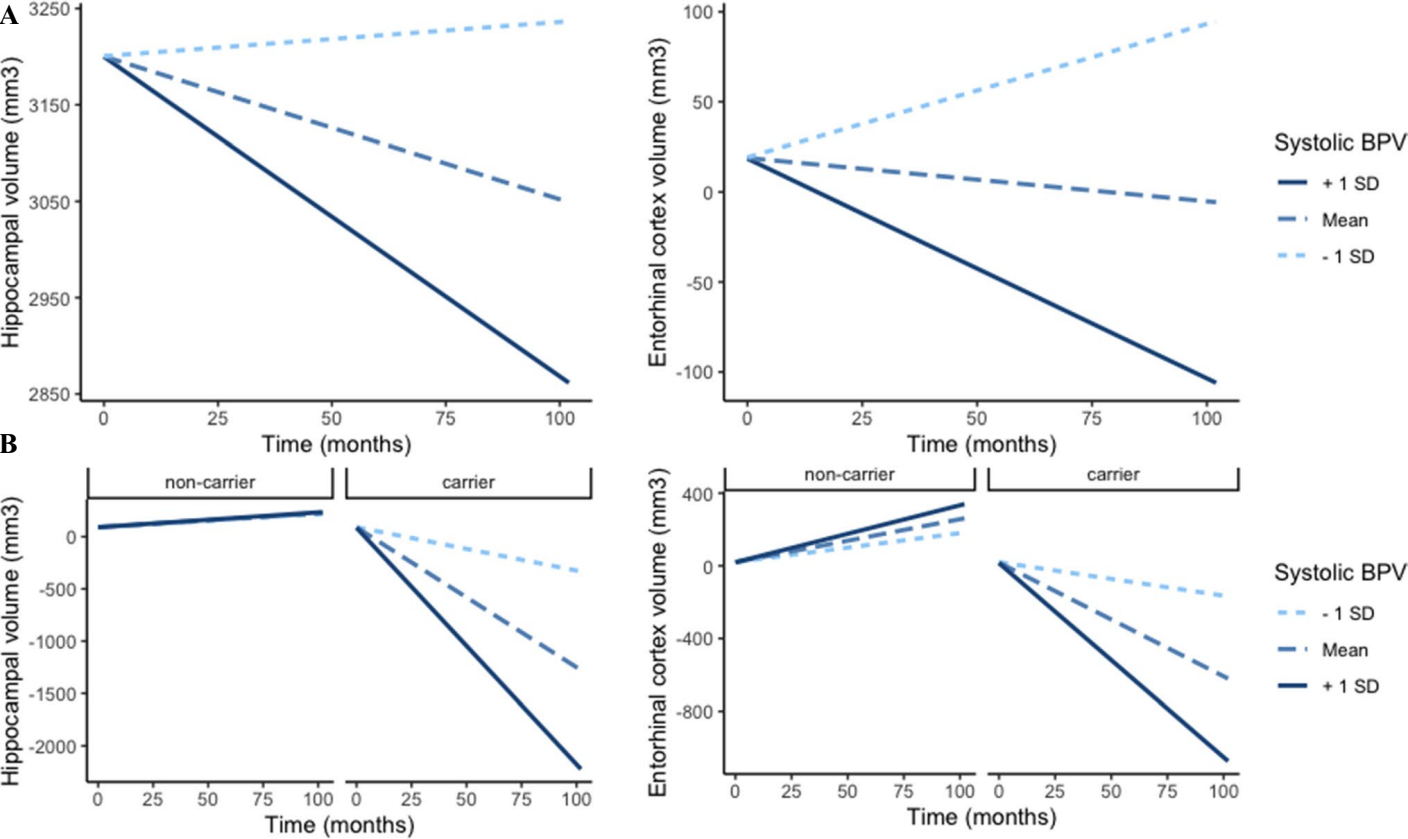
**BPV and medial temporal volumetric change in older adults.** Conditional effects of the interaction of **A)** BPV by time and **B)** BPV by APOE ϵ4 carrier status by time on hippocampal and entorhinal cortex volume in older adults without history of dementia or stroke. Abbreviations: BPV = blood pressure variability

Primary analyses revealed a significant interaction of systolic BPV by time on hippocampal (ß: -0.51 [95% credible interval (CI) -0.63, -0.30]) and entorhinal cortex volume (ß: -0.28 [95% CI -0.34, -0.22]), indicating that participants with elevated systolic BPV were observed to have the fastest hippocampal and entorhinal cortex volume decline at follow-up (Fig. 1a). There was also a significant three-way interaction of systolic BPV by APOE ϵ4 carrier status by time on hippocampal (ß: -2.61 [95% CI -3.02, -2.12]) and entorhinal cortex volume (ß: -1.47 [95% CI -1.71, -1.17]), suggesting that hippocampal and entorhinal cortex volume at follow-up decreased the fastest for APOE ϵ4 carriers with elevated systolic BPV (Fig. 1b).

### BPV and APOE ϵ4 related to medial temporal volumetric change in older adults with AD biomarker abnormalities

**Fig. 2:**
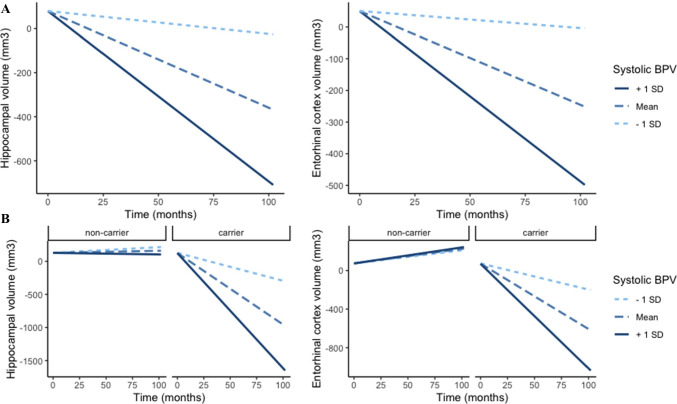
**BPV and medial temporal volumetric change in older adults with AD pathophysiology.** Conditional effects of the interaction of **A)** BPV by time and **B)** BPV by APOE ϵ4 carrier status by time on hippocampal and entorhinal cortex volume in older adults with abnormal levels of both CSF Aβ and Ptau. Abbreviations: BPV = blood pressure variability; AD = Alzheimer’s disease; CSF = cerebral spinal fluid

Secondary analyses of subsets with abnormal levels of both CSF Aβ and Ptau also revealed a significant interaction of systolic BPV by time on hippocampal (ß: -1.05 [95% CI -1.18, -0.36]) and entorhinal cortex volume (ß: -0.78 [95% CI -1.30, -0.56]), suggesting that hippocampal and entorhinal cortex volume change at follow-up was related to elevated systolic BPV in older adults confirmed to have AD pathophysiology (Fig. 2a). Additionally, there was a significant three-way interaction of systolic BPV by APOE ϵ4 carrier status by time on hippocampal (ß: -1.89 [95% CI -2.44, -1.31]) and entorhinal cortex (ß: -1.36 [95% CI -2.04, -0.11]) volume, indicating that volumetric change at follow-up was related to elevated systolic BPV specifically in APOE ϵ4 carriers with AD pathophysiology (Fig. 2b).

Exploratory post-hoc analyses of the three-way interaction of BPV by APOE ϵ4 carrier status by time in subsets not meeting biomarker criteria for AD revealed no significant relationships with medial temporal volume change (see Supplementary Table 2).

Findings were largely consistent in analyses of diastolic BPV (see Supplementary Results).

Primary findings in hippocampal and entorhinal cortex volume change remained significant and essentially unchanged in sensitivity analyses controlling for average BP, history of smoking, history of dyslipidemia, use of antidementia agents, and whole brain volume at MRI (instead of TIV) (Data not shown).

Supplementary analyses revealed a significant interaction of BPV by time on whole brain volume, consistent with one study examining BPV and brain volume change (Ma et al., 2020c). There were no significant interactions with APOE ϵ4 carrier status on whole brain volume (see Supplementary Results).

## Discussion

Findings indicate increased BPV is related to medial temporal volume loss, particularly among APOE ϵ4 carriers, suggesting a potential interplay between genetic susceptibility to medial temporal pathology and systemic hemodynamic dysregulation. The current investigation confirms prior work linking elevated BPV to hippocampal atrophy (Ma et al., 2020c; Sabayan et al., 2013) and extends findings by identifying the key role of APOE ϵ4 in determining the relationship between BPV and both hippocampal and entorhinal cortical atrophy. Additionally, the present study identifies relationships between BPV and medial temporal atrophy in older adults with ongoing AD pathophysiology based on abnormal levels of both CSF Aβ and Ptau, indicating that links between BPV and medial temporal atrophy are implicated in biomarker-confirmed AD. Study findings provide additional insights into the growing body of evidence that BPV is associated with AD and not other neurodegenerative diseases (Lattanzi et al. 2015, 2014a, 2014b).

The link between BPV and volumetric decline was predominantly observed in APOE ϵ4 carriers, a population known to be at increased risk for medial temporal atrophy (Burggren et al., 2008; Palop & Mucke, 2011) and AD (Corder et al., 1993). Specifically, an increase of 1 SD in BPV was associated with a 0.27%—0.33% reduction in hippocampal and entorhinal cortex volume per month (3.3% – 4.0% per year); in APOE ϵ4 carriers, volumes declined by 0.67%—0.70% per month (8.0%—8.4% per year), consistent with prior studies on the association between APOE ϵ4 and hippocampal volume change (Cohen et al., 2001; Jak et al., 2007; Moffat et al., 2000). APOE ϵ4 carriers also display breakdown of the blood–brain barrier (Nation et al., 2019) in the hippocampus and parahippocampal cortex before the onset of cognitive impairment and independent of Aβ and tau pathology (Montagne et al., 2020). It has been proposed that chronic high amplitude oscillations in BP may create a repeated “tsunamic effect” in the cerebral parenchyma (Yoo et al., 2020), distending the arterial walls beyond repair and disrupting the tight junction of the blood–brain barrier. This effect may be especially pronounced in APOE ϵ4 carriers with genetic neurovascular vulnerability in smaller vascular compartments, where unsteady pulsatile forces may exacerbate a leaky blood–brain barrier (Vikner et al., 2021; Winder et al., 2021) and increase the risk for cerebral small vessel disease. It is striking that the current observations were made in a study sample with limited cerebrovascular disease (Hachinski Ischemic score ≤ 4). More studies of individuals with varying levels of cerebrovascular disease burden will help further elucidate the role BPV may play in blood–brain barrier dysfunction and subsequent neurodegeneration. An alternative possibility is that neurodegeneration in APOE ϵ4 carriers may impact cerebral autonomic control of circulation, potentially driving BP fluctuations (Kitamura et al., 2020). Therefore, causal inference of the current investigation is limited, but the longitudinal design indicating BPV predicts future volumetric change after BPV measurement suggests BPV may play a causal role in brain volume loss in APOE ϵ4 carriers. Interestingly, BPV was also related to whole brain volume decline, but this was not significantly related to APOE ϵ4 carrier status. This further highlights the role of BPV in medial temporal atrophy in a population with known regional vulnerability. However, while sensitivity analyses controlling for whole brain volume (instead of TIV) revealed essentially the same pattern of findings, it is important to consider regional volume loss in the context of declining whole brain volume.

In addition to the potential contributions of BPV to cerebrovascular dysfunction, elevated BPV may interfere with vascular clearance mechanisms important for elimination of toxic proteins from the brain (Lattanzi et al., 2018). The observed relationships between BPV and volumetric change were stronger in participant subsets with abnormal levels of CSF Aβ and Ptau and absent in participant subsets not meeting biomarker criteria for AD, suggesting the link between BPV and medial temporal atrophy appears in the context of AD pathophysiology.

Arterial stiffening may amplify BP fluctuations, potentially driving elevated BPV in association with neuronal atrophy (Ma et al., 2020b). While the present investigation was not able to characterize arterial stiffness in the study sample, future work exploring relationships with arterial stiffening may better clarify the contribution of arterial health on brain volume decline. Relatedly, investigating the role of BPV in patterns of brain atrophy as a vascular mechanism linking BPV to dementia risk may have therapeutic implications. Emerging evidence suggests that some classes of antihypertensive medication have differential effects on the variability of BP for risk of stroke, independent of average levels (Webb et al., 2010). Large studies adequately powered to investigate treatment effects on BPV, for antihypertensive monotherapy as well as for combination therapy, may lead to additional therapeutic strategies beyond aggressive BP control for the prevention of cognitive impairment. Importantly, BP is a highly modifiable risk factor for dementia (Barnes & Yaffe, 2011) and even slight changes in BP control may have large public health implications for both cardiovascular and cognitive outcomes (Yaffe, 2019).

The present investigation has a number of strengths. First, by investigating volumetric change after the measurement of BPV, we were able to appreciate the temporal order of the role of BPV and the possibly synergetic effects of BPV and APOE ϵ4 on volumetric change in brain regions implicated in early AD pathology. Second, to further highlight the possibility of BPV as a vascular risk factor in the context of AD, we utilized participant subsets who were confirmed to have AD pathophysiology based on CSF measurement of AD biomarkers Aβ and Ptau.

The study has several noteworthy limitations. Some aspects of BP collection were not explicitly standardized across sites. Additionally, the ADNI database is largely comprised of non-Hispanic White older adults; thus, generalizability of findings to other racial and ethnic groups is limited. There is mixed evidence for whether the relationship between BPV and cerebrovascular disease may differ by race or ethnicity (Brickman et al., 2010; Tully et al., 2020). Studies involving more diverse samples will help to understand potential differences. As part of the inclusionary enrollment criteria for ADNI, the current study did not include older adults with more extensive cerebrovascular disease. Increased BPV has been associated with cerebrovascular damage, which may act as a potential confounder of brain atrophy (Ma et al., 2020a; Sible et al., 2021a; Tully et al., 2020) and contribute to biomarker evidence of AD, possibly independent of APOE ϵ4. Studies that include samples with varying levels of cerebrovascular disease severity will help clarify the relationship between BPV, cerebrovascular damage, and neurodegeneration.

## Conclusions

Elevated BPV in older adults was related to brain volumetric change over time in regions implicated in AD dementia and pathology, independent of baseline hypertension. APOE ϵ4 moderated this relationship, suggesting a potentially synergetic effect of both elevated BPV and APOE ϵ4 on volumetric decline. Finally, patterns of decline were observed to be strongest in individuals with AD pathophysiology, which may implicate BPV as an understudied aspect of vascular contributions to dementia.

## Supplementary Information

Below is the link to the electronic supplementary material.

Supplementary file1 (DOCX 22 kb)

## Data Availability

The datasets generated and/or analyzed during the current study are available in the ADNI database, https://adni.loni.usc.edu.

## Code availability

All analyses were conducted using R.
