# Supplementary material for: Blood pressure variability and medial temporal atrophy in apolipoprotein ϵ4 carriers
Source: Brain Imaging Behav. 2021 Sep 28;16(2):792–801. doi: 10.1007/s11682-021-00553-1 (PMC9009865; doi:10.1007/s11682-021-00553-1)
Supplement: Supplementary file 1 — Supplementary file1 (DOCX 22 kb) [file 11682_2021_553_MOESM1_ESM.docx]

Supplementary Materials

RESULTS

*Diastolic BPV analyses*

BPV and APOE ϵ4 related to medial temporal volumetric change in older adults

Primary analyses of older adults without history of dementia or stroke revealed a significant interaction of diastolic BPV by time on hippocampal (ß: -.56 [95% credible interval (CI) -.70, -.42]) and entorhinal cortex volume (ß: -.25 [95% CI -.29, -.22]), indicating that participants with elevated diastolic BPV were observed to have the fastest hippocampal and entorhinal cortex volume decline (Data not shown). There was also a significant three-way interaction of BPV by APOE ϵ4 carrier status by time on hippocampal (ß: -2.41 [95% CI -2.76, -1.72]) and entorhinal cortex volume (ß: -1.60 [95% CI -1.93, -1.27]), suggesting that hippocampal and entorhinal cortex volume decreased the fastest for APOE ϵ4 carriers with elevated diastolic BPV (Data not shown).

BPV and APOE ϵ4 related to medial temporal volumetric change in older adults with AD biomarker abnormalities

Secondary analyses of participant subsets with abnormal levels of both CSF Aβ and Ptau also revealed a significant interaction of diastolic BPV by time on hippocampal (ß: -1.42 [95% CI -1.53, -.89]) and entorhinal cortex volume (ß: -.96 [95% CI -1.37, -.89]), suggesting that hippocampal and entorhinal cortex volume change over time was related to elevated diastolic BPV in older adults confirmed to have AD pathophysiology (Data not shown). The three-way interaction of diastolic BPV by APOE ϵ4 carrier status by time in those with AD pathophysiology was not significant for hippocampal (ß: -1.38 [95% CI -1.69, .31]) or entorhinal cortex volume (ß: -1.53 [95% CI -2.36, 0.19]) (Data not shown).

*Whole brain volume*

Primary analyses of older adults without history of dementia or stroke revealed a significant interaction of BPV by time on whole brain volume (systolic: ß: -.14 [95% CI -.16, -.08]; diastolic: ß: -.24 [95% CI -.26, -.21]) (Data not shown). No significant interactions emerged with APOE ϵ4 carrier status (systolic: ß: .04 [95% CI -.86, .33]; diastolic: ß: .06 [95% CI -.08, .19]) (Data not shown).

**Supplementary Table 1.**

Sample sizes and MRI scan characteristics for post-hoc exploratory analyses of participant subsets not meeting biomarker criteria for AD

| **Subset** | ***n*** | **Total # scans** | **Median # scans per participant** | **Median (IQR) time interval between BPV measurement and scan** |
| --- | --- | --- | --- | --- |
| Aβ+Ptau- or Aβ-Ptau+ | 266 | 605 | 2 | 24 |
| Aβ-Ptau- | 253 | 525 | 3 | 24 |

Abbreviations: Aβ = amyloid-beta; Ptau = phosphorylated tau

**Supplementary Table 2.**

Estimates of BPV by APOE ϵ4 carrier status by time interaction in post-hoc exploratory participant subsets not meeting biomarker criteria for AD

|  | **ß (95% credible interval)** | |
| --- | --- | --- |
| **Subset** | **Systolic BPV** | **Diastolic BPV** |
| Aβ+Ptau- or Aβ-Ptau+ |  |  |
| Hippocampus | -.37 (-1.12, .40) | -.40 (-1.22, .40) |
| Entorhinal cortex | -.41 (-1.19, .35) | -.38 (-1.17, .39) |
| Aβ-Ptau- |  |  |
| Hippocampus | -.41 (-1.20, .39) | -.36 (-1.14, .42) |
| Entorhinal cortex | -.37 (-1.26, .40) | -.42 (-1.25, .40) |

Models adjusted for age at MRI scan, sex, years of education, TIV, baseline hypertension, antihypertensive medication use and vascular risk.

Abbreviations: BPV = blood pressure variability; Aβ = amyloid-beta; Ptau = phosphorylated tau

**Supplementary Table 3.**

BP information for biomarker subsets based on APOE ϵ4 carrier status

| **Subset** | **Average systolic BP** | **Systolic BPV VIM** |
| --- | --- | --- |
| Meets biomarker criteria for AD |  |  |
| APOE ϵ4- (*n* = 73) | 136.5 (14.5) | 5.8 (3.7) |
| APOE ϵ4+ (*n* = 179) | 132.7 (12.2) | 5.4 (3.2) |
| Does not meet biomarker criteria for AD |  |  |
| APOE ϵ4- (*n* = 391) | 132.4 (12.1) | 5.4 (3.8) |
| APOE ϵ4+ (*n* = 128) | 132.3 (15.1) | 5.3 (3.3) |

Abbreviations: BP = blood pressure; BPV = blood pressure variability;VIM = variability independent of mean; AD = Alzheimer’s disease; APOE ϵ4 = apolipoprotein ϵ4; Aβ = amyloid-beta; Ptau = phosphorylated tau
